# Novel Approach to Increasing the Amplitude of the Mechanical Oscillations of Self-Oscillating Gels: Introduction of Catalysts Both as Pendant Groups and as Crosslinkers

**DOI:** 10.3390/gels10110727

**Published:** 2024-11-09

**Authors:** Ilya L. Mallphanov, Michail Y. Eroshik, Dmitry A. Safonov, Alexander V. Sychev, Vyacheslav E. Bulakov, Anastasia I. Lavrova

**Affiliations:** 1 Center for Nonlinear Chemistry, Immanuel Kant Baltic Federal University, 14 A. Nevskogo Street, Kaliningrad 236016, Russia; misha.eroshik@yandex.ru (M.Y.E.); dasafonoff@gmail.com (D.A.S.); aurebours@googlemail.com (A.I.L.); 2 Research Center for Condensed Matter Physics, Kursk State University, 33 Radishcheva Street, Kursk 305000, Russia; sychev1113@gmail.com; 3 Department of General and Bioorganic Chemistry, Pavlov First Saint-Petersburg State Medical University, 6-8 L’va Tolstogo Street, Saint-Petersburg 197022, Russia; bulakov_v@mail.ru; 4 Saint-Petersburg State Research Institute of Phthisiopulmonology, 2-4 Ligovsky Avenue, Saint-Petersburg 191036, Russia

**Keywords:** ruthenium complexes, iron complexes, polymers, self-oscillating gels, Belousov–Zhabotinsky reaction

## Abstract

For the first time, we introduced chemomechanical self-oscillating poly(N-isopropylacrylamide)-based gels containing catalytically active Fe or Ru complexes both as crosslinkers and as pendant groups. All the obtained gels exhibited sustained autonomous oscillations driven by the Belousov–Zhabotinsky reaction within their structure. The Ru complex-based gels also demonstrated pronounced chemomechanical oscillations; they periodically swelled/shrunk when the catalyst was reduced/oxidized. It was found that the combination of catalytically active cross-linking and pendant Ru complexes in the same gel led to a change in the structure of the gel and a significant increase in the amplitude of its mechanical oscillations. The proposed approach allowed for increasing the amplitude of the mechanical oscillations of self-oscillating gels and opened up new possibilities for adjusting their characteristics. We believe that these gels hold potential for the development of soft actuators and systems capable of signal processing through propagating and interacting chemical waves.

## 1. Introduction

Recent advances in the synthesis and exploration of self-oscillating chemomechanical gels, driven by the oscillatory Belousov–Zhabotinsky (BZ) reaction, have paved the way for creating dynamic soft materials [1,2]. These materials, structured from a polymer network immersed in liquid, autonomously exhibit rhythmic motion, providing a foundation for developing innovative systems such as autonomous actuators, fluid transport devices, and chemical signal processors [3,4,5]. Their capacity for producing complex movements [6], such as peristaltic and ciliary actions, offers solutions where traditional mechanical systems fall short.

To create self-oscillating gels, researchers integrate Belousov–Zhabotinsky (BZ) reaction catalysts, such as ruthenium or iron complexes, into poly(N-isopropylacrylamide) gels. Two successful approaches to catalyst integration in a polymer gel matrix have been described in the scientific literature. In the first approach—the most common—the catalyst is incorporated into the linear poly(N-isopropylacrylamide) chains as a covalently attached pendant group, and the linear chains are crosslinked into the gel’s network using a catalytically inactive crosslinker, which is usually N,N′-methylenebisacrylamide [7,8,9].

The BZ reaction itself involves a series of chemical transformations, but its main steps include the bromination and oxidation of organic compounds, like malonic acid, in the presence of bromate and catalytically active ruthenium or an iron complex within an acidic solution [10,11]. As the reaction proceeds, the catalyst undergoes periodic changes in its oxidation state. When the catalyst is covalently attached to the gel’s polymer network, these changes lead to cyclic variations in the physical characteristics of the gel, such as color, size, and transparency.

When a crosslinked poly(N-isopropylacrylamide) gel with a covalently attached ruthenium or iron-based catalyst is placed in a catalyst-free BZ (CFBZ) solution, an autonomous oscillatory redox reaction occurs in the CFBZ–gel system. This reaction triggers periodic redox transitions in the catalyst. In the case of poly(N-isopropylacrylamide) gels, these changes in the oxidation state of the catalyst alter the volume phase transition temperature and the gel’s swelling behavior, as the polymer chains become more hydrophilic during oxidation and less hydrophilic during reduction [12]. Consequently, the gel exhibits periodic swelling when the catalyst is oxidized and contraction when it is reduced, resulting in autonomous chemomechanical oscillations that synchronize with the redox potential fluctuations of the catalyst. The gels obtained using the first approach and containing pendant catalytically active ruthenium complexes occupy a dominant position in the scientific literature [13,14,15]; similar gels containing catalytically active pendant iron complexes are much less common [8,9,16,17].

In the second, less common approach, the metal complex within the gel simultaneously functions as both a catalyst and a crosslinking agent, effectively acting as an “active crosslinker”. This behavior parallels biological systems, such as the myosin motors in muscle cells that connect actin filaments [18]. Inspired by this mechanism, which converts chemical energy into mechanical motion, researchers are working to create autonomous, self-oscillating gels that mimic the behavior of muscle tissue [19,20].

For example, the authors in [19] utilized a tris-crosslinker Ru complex, [Ru(L)_3_^2+^, where L = N,N′-diallyl-(2,2′-bipyridine)-4,4′-dicarboxamide], to crosslink poly(N-isopropylacrylamide) chains. In this case, in obtained gel, each ligand connects two polymer chains, and each Ru atom coordinates with three ligands. This gel demonstrates chemomechanical oscillations during the BZ reaction.

The authors in [20] synthesized Ru and Fe complex crosslinked gels utilizing a mono-crosslinker [RuL(bpy)_2_^2+^, where L = bis(4-vinylbenzyl)[2,2′-bipyridine]-4,4′-dicarboxylate or N4,N4′-bis(4-vinylphenyl)-2,2′-bipyridine-4,4′-dicarboxamide] or tris-crosslinker [FeL_3_^2+^, where L is identical to the Ru complex ligand] to crosslink poly(N-isopropylacrylamide) chains. In this work, the chemomechanical properties of the Fe complex-crosslinked gels were not confirmed experimentally; the Ru complex-crosslinked gels exhibited mechanical oscillations.

In summary, there are numerous examples of self-oscillating gels (crosslinked with inactive crosslinkers) containing pendant catalysts and a few examples of gels crosslinked with catalytically active complexes, but there are no examples of gels having catalytically active fragments both as crosslinkers and as pendant groups.

Furthermore, to date, only Ru complex-crosslinked self-oscillating gels had been synthesized and evaluated in the BZ reaction. The development of self-oscillating gels crosslinked with Fe complexes and their subsequent evaluation in the BZ reaction remained an unresolved challenge. It is important to highlight that Ru complexes possess notable drawbacks, including high cost, complex synthesis procedures, and sensitivity to light. The creation of affordable, robust, and easily synthesized self-oscillating gels based on Fe catalysts, including Fe catalyst-crosslinked gels, remains a critical objective, since for practical industrial use it is necessary to develop cheap, accessible chemomechanical materials, which is satisfied by Fe catalyst-based gels, but not by expensive Ru catalyst-based gels.

The first objective of our study was to create novel Fe and Ru catalyst-crosslinked self-oscillating gels based on new Fe or Ru complexes that simultaneously act as catalysts and crosslinkers, to test the resulting gels under the conditions of the BZ reaction and compare their self-oscillating behavior and chemomechanical properties with the properties of self-oscillating gels containing pendant catalytically active complexes of the same metals, but crosslinked with inactive N,N′-methylenebisacrylamide. We synthesized (N4,N4′-bis[(3-(2-methylprop-2-enamido)propyl][2,2′-bipyridine]-4,4′-dicarboxamide)bis(2,2′-bipyridine)iron(II) sulfate (2) and (N4,N4′-bis[(3-(2-methylprop-2-enamido)propyl][2,2′-bipyridine]-4,4′-dicarboxamide)bis(2,2′-bipyridine)ruthenium(II) chloride (3) and copolymerizing (2) or (3) with N-isopropylacrylamide-obtained Fe or Ru catalyst-crosslinked gels (7) or (8), correspondingly. Gel (7) demonstrated self-oscillating behavior without mechanical oscillations; gel (8) periodically swelled when the catalyst was oxidized and shrunk when it was reduced. The self-oscillating behavior of the Fe catalyst-crosslinked gel (7) was demonstrated for the first time.

To obtain gels with pendant catalysts, we synthesized (5-acrylamido-1,10-phenanthroline)bis(1,10-phenanthroline)iron(II) sulfate (5) and (5-acrylamido-1,10-phenanthroline)bis(2,2′-bipyridine)ruthenium(II) chloride (6). These compounds were then copolymerized with N-isopropylacrylamide and N,N′-methylenebisacrylamide to produce gels (9) and (10), respectively. The resulting gels exhibited self-oscillatory behavior, characterized by periodic swelling during the oxidation of the catalyst and subsequent shrinking upon reduction. A comparative analysis of the self-oscillatory behavior and the chemomechanical properties of gels (7) and (8) was conducted alongside gels (9) and (10). This part of our study highlights the novelty of creating and evaluating new Fe and Ru catalyst-crosslinked gels and comparing their self-oscillatory behaviors with those of gels containing pendant catalysts at equivalent concentrations.

The second goal of our study was to test a novel approach for enhancing the amplitude of chemomechanical oscillations in gels. This strategy involved incorporating catalytically active fragments both as crosslinks and as pendant elements within the linear polymer chain. Using this approach, we synthesized self-oscillating gel (11) by copolymerizing (2) and (5) with N-isopropylacrylamide, and synthesized gel (12) by copolymerizing (3) and (6) with N-isopropylacrylamide. The innovativeness of the adopted solution in comparison to the already existing ones is that this approach produced a gel that was very different in microstructure and elastic modulus from both the catalyst-crosslinked gels and the gels with pendant catalysts. This made it possible to significantly increase the amplitude of the mechanical oscillations (change in linear size reaches 16%) in the case of gel (12).

## 2. Results and Discussion

### 2.1. Gels Crosslinked with Catalysts (2) and (3)

**Figure 1:**
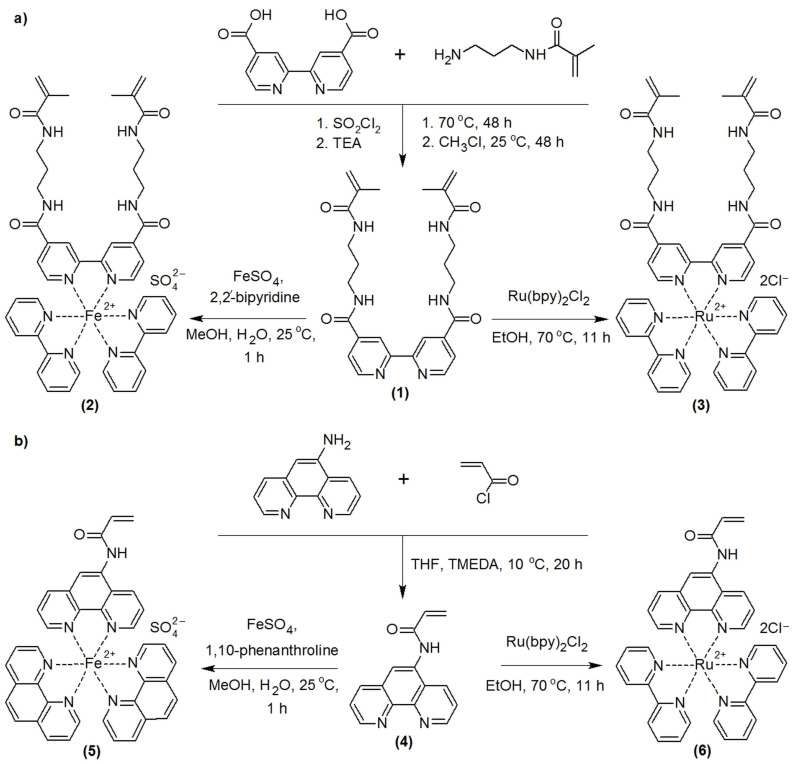
(**a**) Synthesis of ligand (**1**) and catalysts (**2**) and (**3**). (**b**) Synthesis of ligand (**4**) and catalysts (**5**) and (**6**). TEA—triethylamine, TMEDA—tetramethylethylenediamine, Ru(bpy)_2_Cl_2_—bis(2,2′-bipyridine)ruthenium(II) chloride, THF—tetrahydrofuran, MeOH—methanol, EtOH—ethanol.

Initially, we synthesized crosslinker N4,N4′-bis[(3-(2-methylprop-2-enamido) propyl)] [2,2′-bipyridine]-4,4′-dicarboxamide (1) (Figure 1a, Materials and Methods, Section 4.2), which incorporated a 2,2′-bipyridine unit designed for complexation with metal ions (Fe^2+^ or Ru^2+^) and two polymerizable methacrylamide groups, linked to the bipyridine core via spacers. This particular ligand was selected due to its structural advantages over those used in previous studies [19,20]. The inclusion of spacers helped to distance the methacrylamide groups from the 2,2′-bipyridine fragment, thereby reducing the steric hindrance during the copolymerization of the complexes [formed from (1)] with other monomers. Subsequently, we synthesized the Fe catalyst–crosslinker (2) (Figure 1a) by reacting one equivalent of (1) with one equivalent of iron(II) sulfate and two equivalents of 2,2′-bipyridine, as outlined in Materials and Methods, Section 4.3.

To obtain a Ru catalyst–crosslinker, we synthesized complex (3) via the reaction of (1) with bis(2,2′-bipyridine)ruthenium(II) chloride (Figure 1a, Materials and Methods, Section 4.4). We decided to abandon the approach of utilizing Fe and Ru catalyst–crosslinkers that incorporated three ligands (1) and were capable of forming six bonds with polymer chains, as previously suggested by other researchers [19]. This decision was made due to the concern that, under the conditions of mechanical oscillations, the Fe or Ru ligand bonds in such complexes would experience excessive stress, leading to potential bond rupture. In contrast, in complexes (2) and (3), ligand (1) occupied two coordination sites of the metal ion, thereby functioning as a mono-crosslinker, while the remaining four coordination sites were filled by 2,2′-bipyridine molecules, which were inert in the polymerization process.

**Figure 2:**
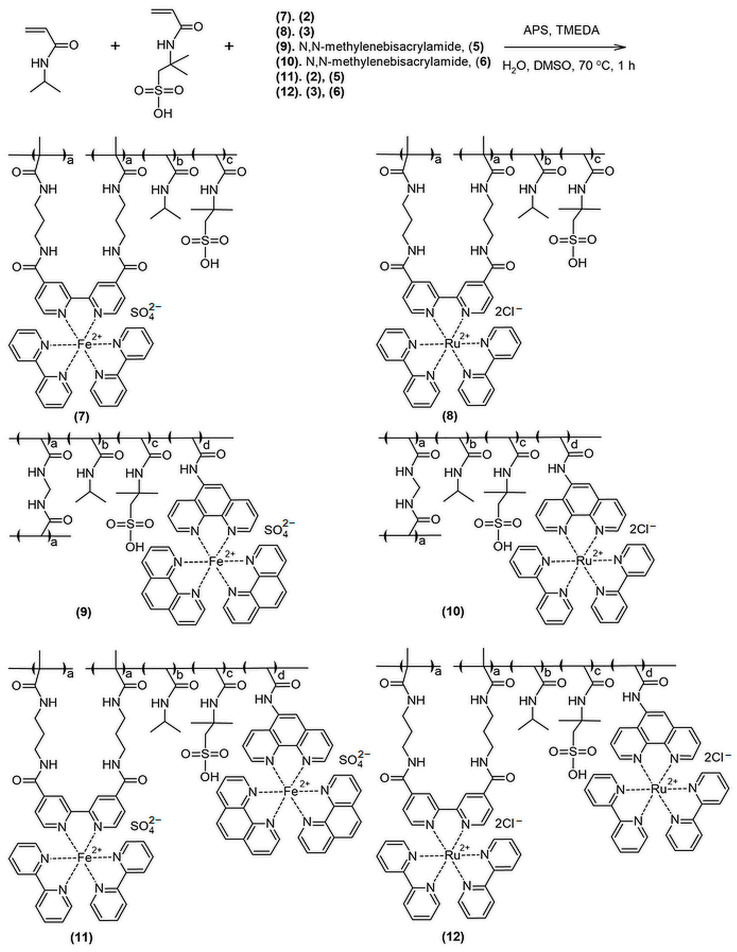
APS—ammonium persulfate; TMEDA—tetramethylethylenediamine; DMSO—dimethyl sulfoxide.

Copolymerizing the complexes (2) and (3) with N-isopropylacrylamide (IPA) and 2-acrylamido-2-methylpropanesulfonic acid (AMPS), we synthesized gels (7) and (8) (Figure 2, Materials and Methods, Section 4.8) correspondingly. In the gels, the complexes (2) and (3) were used as catalytic and mono-crosslinking fragments; IPA and AMPS were introduced as monomers forming a polymer chain and microphase-separated structure, respectively.

Gels (7) and (8) were synthesized as 0.5 cm^3^ pieces. After soaking them in distilled water to remove unreacted low-molecular compounds, gels were obtained as red (7) and orange (8) materials. The estimated catalyst concentrations in gels (7) and (8) were 0.93 and 1.02 mole% relative to IPA, correspondingly, which was determined from the difference between the amount of the catalyst introduced during synthesis and washed out during gel soaking (Materials and Methods, Section 4.8). The obtained gels were cut into pieces smaller than a millimeter for studying of the oscillatory properties, since larger pieces, due to the low speed of propagation of their chemical wave, can have both oxidizing (expanding) and reducing (contracting) regions simultaneously. To test the chemomechanical properties of gels (7) and (8), the pieces were immersed in an aqueous CFBZ solution: [MA] = 0.063 M, [NaBrO_3_] = 0.084 M, [HNO_3_] = 0.9M.

**Figure 3:**
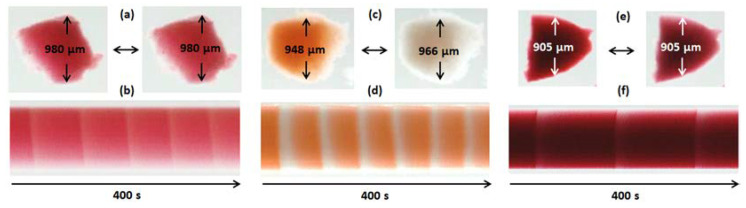
Oscillations of the pieces of the gels in the CFBZ solution with the following concentrations: [MA] = 0.063 M, [NaBrO_3_] = 0.084 M, [HNO_3_] = 0.9 M. (**a**,**c**,**e**) Snapshots of the pieces of gels (7), (9) and (11), respectively, in the reduced (Fe^2+^) (left snapshot) and in the oxidized (Fe^3+^) (right snapshot) states of the complexes. (**b**,**d**,**f**) Space–time plots of the pieces of gels (7), (9) and (11), respectively. The uncertainty of the size measurement is 1 μm.

A piece of gel (7) (Figure 3a) shows the self-oscillatory behavior in the CFBZ solution becoming pink (red) in the oxidized (reduced) state of the catalyst but not changing its size during the oxidation–reduction transitions of the complex.

Figure 3b shows the space–time plot of the oscillations of a piece of gel (7). The space–time plot demonstrates the color of the piece as a function of time. The experimental setup used for recording the snapshots of the pieces of the gels and measuring their sizes as well as a procedure for building space–time plots are given in Materials and Methods, Section 4.9.

**Figure 4:**
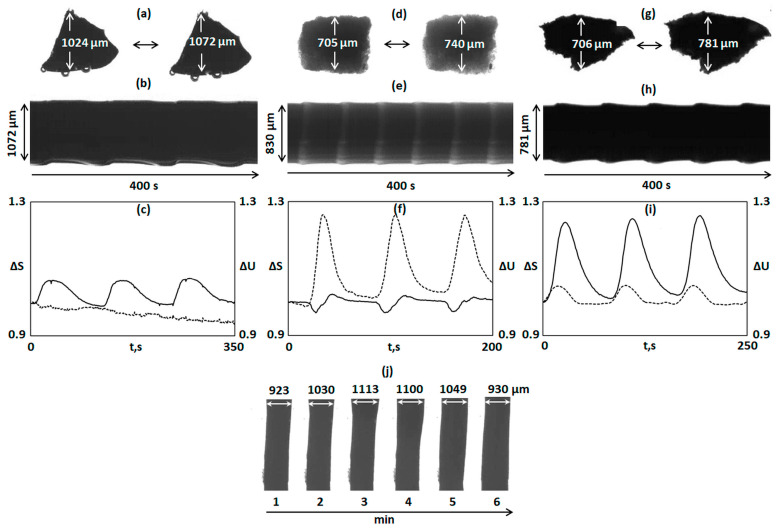
Oscillations of the pieces of the gels in the CFBZ solution with the following concentrations: [MA] = 0.063 M, [NaBrO_3_] = 0.084 M, [HNO_3_] = 0.9 M. (**a**,**d**,**g**) Snapshots of the pieces of gels (8), (10), and (12), respectively, in the reduced (Ru^2+^) (left snapshot) and in the oxidized (Ru^3+^) (right snapshot) states of the complexes. (**b**,**e**,**h**) Space–time plots of the pieces of gels (8), (10), and (12), respectively. (**c**,**f**,**i**) Dependence of ∆S = S_i_/S_0_ (solid line) and ∆U = U_i_/U_0_ (dotted line) on the piece on the time (t,s) for gels (8), (10), and (12), correspondingly. (**j**) Snapshots of gel (12). The cylindrical gel (diameter 1 mm, height 3.6 mm) is immersed in the aqueous CFBZ solution. The chemical wave propagates in the gel from top to bottom at a speed of about 0.6 mm/min in the direction of the gel length; the locally swollen and shrunken parts move with the chemical wave. The uncertainty of the size measurement is 1 μm.

A piece of gel (8) exhibits noticeable chemomechanical oscillations in the CFBZ solution (Figure 4a). The size of the piece increased when the catalyst was in the oxidized state and decreased when it was in the reduced state; the mechanical oscillations of the gel coincided with the chemical ones. The amplitude of the mechanical oscillations (the alternations in the linear size of the gel) was about 3.5%. Figure 4a shows snapshots of the shrunken (left snapshots) and swollen (right snapshot) states of a piece of gel (8) and Figure 4b shows a space–time plot of its dynamics. The space–time plot demonstrates the size and color of the piece as a function of time. Since gel is almost opaque, it is difficult to observe chemical oscillations in the CFBZ solution via the optical method, but mechanical oscillations are clearly visible.

Here and below, the oscillations of the Fe complex-based gels were observed through a microscope with a color camera and white light illumination; the oscillations of the Ru complex-based gels were observed through a microscope with a black-and-white camera and white light illumination through an interference filter with the wavelength of the maximum transmission at λ = 450 nm; since Ru complex-based gels are light-sensitive and may not oscillate when illuminated with white light.

### 2.2. Gels Crosslinked with MBA and Containing Pendant Catalysts (5) and (6) in Linear Polymer Chains

To compare the chemomechanical behavior of the obtained gels (7) and (8) with the gels containing the same concentrations of pendant Fe or Ru catalysts in the linear chains and crosslinked with catalytically inactive MBA, we synthesized catalysts (5) and (6), which were incapable of crosslinking polymer chains but were capable of being incorporated into them. First, we synthesized 5-acrylamido-1,10-phenanthroline (4) (Figure 1b, Materials and Methods, Section 4.5), which contains a 1,10-phenanthroline moiety for complexation with metals ions (Fe^2+^ or Ru^2+^) and one polymerizable acrylamide group. Next, we synthesized the Fe complex (5) (Figure 1b, Materials and Methods, Section 4.6) by combining one equivalent of (4) with one equivalent of iron(II) sulfate and two equivalents of 1,10-phenanthroline, and also Ru complex (6) via the reaction of (4) with bis(2,2′-bipyridine)ruthenium(II) chloride (Figure 1b, Materials and Methods, Section 4.7).

The copolymerization of (5) and (6) with IPA, AMPS, and MBA yielded gels (9) and (10) (see Materials and Methods, Section 4.8), respectively. In gels (9) and (10), complexes (5) and (6) were used as catalytic fragments; MBA was introduced as a crosslinking agent; IPA and AMPS were used as monomers forming a polymer chain and microphase-separated structure, respectively. After soaking the gels in water to remove their unreacted compounds, they were obtained as red (9) and orange (10) materials. The estimated catalysts’ concentration in gels (9) and (10) were 1.05 and 1.07 mole% relative to IPA, correspondingly, which was determined from the difference between the amount of the catalyst introduced during synthesis and washed out during gel soaking (Materials and Methods, Section 4.8).

We tested gels (9) and (10) in the CFBZ mixture under the same conditions as gels (7) and (8). In contrast to gel (7), gel (9) exhibited small-amplitude chemomechanical oscillations (Figure 3c,d); the changes in the linear size of the gel were about 2%. Gel (10) showed chemomechanical oscillations with an amplitude of about 4.5% (Figure 4d,e), which was close to the value (3.5%) obtained for gel (8). Thus, for the gels based on the Ru complexes, it can be assumed that at the same concentration of IPA and AMPS in the gels, as well as at the same crosslinking (1 mole% relative to IPA) of the gel, it was the concentration of the catalyst that mostly determined the amplitude of the chemomechanical oscillations of the gel, regardless of whether the catalyst played the role of crosslinking or was built into the linear chains of the polymer.

### 2.3. Gels Crosslinked with Catalysts (2) and (3) and Containing Pendant Catalysts (5) and (6) in Linear Polymer Chains

To increase the amplitude of the mechanical oscillations, we created gels containing catalysts both as crosslinkers and as pendant components of the linear polymer chain (see Materials and Methods, Section 4.8). We synthesized the following: gel (11), containing (2) as a crosslinker and (5) as a pendant fragment; gel (12), containing (3) as a crosslinker and (6) as a pendant fragment. The estimated catalysts’ concentration in gels (11) and (12) were 1.82 and 1.90 mole% relative to IPA, correspondingly, which was determined in the same way as in the case of the previous gels (Materials and Methods, Section 4.8).

In the aqueous CFBZ solution, a piece of gel (11) (Figure 3e,f) exhibited periodic color changes but no chemomechanical oscillations. The piece turned pink when the catalysts (2) and (5) were in the oxidized state (Fe^3+^) and turned red when the catalysts were in the reduced state (Fe^2+^). The reason for the absence of the chemomechanical oscillations despite the high total content of the catalysts in the gel is not yet clear to us, and the behavior of such “heterocatalyst” gels requires further study.

In contrast to gel (11), a piece of gel (12) demonstrated high-amplitude chemomechanical oscillations in the aqueous CFBZ solution (Figure 4g,h). The periodic change in the linear size of the piece of gel (12) was about 11%. The oscillatory behavior of gel (12) is demonstrated in the Movie S1 (see Supplementary Material).

Gel (12) also was synthesized in the form of a cylinder (diameter about 1 mm, height about 3.6 mm). When the cylinder was immersed in an aqueous CFBZ solution, a chemical wave arose and propagated in it at a speed of about 0.6 mm/min in the direction of the gel length; the locally swollen and shrunken parts moved with the chemical wave, like the peristaltic motion of living worms (Figure 4j). The periodic change in the diameter of the cylinder of gel (12) was about 16%. The oscillatory behavior of the cylinder of the self-oscillating gel 12 is demonstrated in the Movie S2 (see Supplementary Material).

### 2.4. Discussion

We assumed that in the case of using both the Fe and Ru catalysts, the catalytic cycle is realized as described earlier [21]:

BrO_3_^−^ + Br^−^ + 2H^+^ ⇄ HBrO_2_ + HOBr

HBrO_2_ + Br^−^+ H^+^ ⇄ 2HOBr

BrO_3_^−^ + HBrO_2_ + H^+^ ⇄ 2BrO_2_^•^ + H_2_O

BrO_2_^•^ + M(L_3_)^2+^ + H^+^ ⇄ HBrO_2_ + M(L_3_)^3+^

2HBrO_2_ ⇄ BrO_3_^−^ + HOBr + H^+^

M(L_3_)^3+^ + BrMA → Br^−^+ M(L_3_)^2+^ + stable products.

The swelling/shrinking of the poly(N-isopropylacrylamide) gels during the BZ reaction was attributed mainly to the changes in the oxidation state of the metal complex M(L_3_) bound with a polymer matrix. The oxidation [M(L_3_)^2+^→M(L_3_)^3+^] of the complex had a hydrating effect and the gel absorbed liquid and swelled; the reduction [M(L_3_)^3+^→M(L_3_)^2+^] of the complex dehydrated the gel and it pushed the liquid into the environment and shrunk [22].

The incorporation of a BZ reaction catalyst within the polymer matrix of the gel was essential for the manifestation of autonomous periodic chemical and mechanical oscillations. In the absence of this catalyst, the gels do not exhibit self-oscillatory behavior; instead, they can only undergo property changes (such as size and transparency) in response to external stimuli, including temperature and pH. It is noteworthy that gels with a microheterogeneous porous structure demonstrate significantly greater amplitudes of chemomechanical oscillations compared to those with a homogeneous structure [23]. To enhance the microheterogeneity of the gel structures, 2-acrylamido-2-methylpropanesulfonic acid (AMPS) was incorporated as an additive in all the gel formulations.

All the Fe catalyst-based gels [(7), (9), (11)] showed self-oscillatory behavior, but only gel (9) containing a pendant catalyst demonstrated low-amplitude chemomechanical oscillations (2%). The self-oscillating behavior of Fe catalyst-crosslinked gel (7) was demonstrated for the first time. The combination of crosslinking (2) and pendant (5) Fe catalysts in gel (11) did not produce a gain in the amplitude of the chemomechanical oscillations. Gel (7) showed no chemomechanical oscillations at 0.93 mole% (relative to IPA) catalyst (2) concentration; gel (9) showed the 2% amplitude at near the same (1.05 mole% relative to IPA) concentration of (5), but its combination in gel (11) resulted in a gel that showed no chemomechanical oscillations despite the high total catalyst content (1.82 mole% relative to IPA) in the gel. This behavior of gel (11) requires further studies.

All the Ru catalyst-based gels [(8), (10), (12)] showed self-oscillatory behavior and pronounced chemomechanical oscillations. Based on the data obtained, we can draw conclusions about the synergetic catalytic effect that occurs when the crosslinking catalyst (3) and the pendant catalyst (6) are combined. This combination of the catalysts in the gel provided a greater gain in the amplitude of the chemomechanical oscillations than the simple summation of the contributions of each catalyst. Gel (8) showed an amplitude of 3.5% at the 1.02 mole% (relative to IPA) concentration of (3) and gel (10) showed a 4.5% amplitude at the 1.07 mole% (relative to IPA) concentration of (6), but its combination in gel (12) did not result in the simple summation of the amplitudes when summing the catalyst concentrations; the resulting amplitude was not 8% but about 11% in the case of gel (12) in the form of a piece, and about 16% in the case of gel (12) in the form of a cylinder.

To determine the reasons for this phenomenon, we compared the graphs of the chemical (changes in the oxidation state of the catalyst) and mechanical (changes in the area) oscillations of the pieces of gels (8) (Figure 4c), (10) (Figure 4f), and (12) (Figure 4i) (see Materials and Methods, Section 4.10). ∆S = S_i_/S_0_ was the ratio of the area of a piece of gel at a given moment (i) in time to the area at the initial (0) moment in time; ∆U = U_i_/U_0_ was the ratio of the shade of a piece of gel at a given (i) moment in time to shade at the initial (0) moment in time. For gel (8), chemical oscillations were poorly visible due to the fact that the gel was not transparent and changes in its shade were difficult to record. For gel (10), chemical and mechanical oscillations were well-visible and the phase difference [∆ϕ = (t_m_ − t_ch_)/T; t_m_—the moment in time at which the gel area is maximum; t_ch_—the moment in time at which the gel has the lightest shade (oxidized state of catalyst); T—period, s] between them was about 0.15; the chemical oscillations occurred first, followed by the mechanical oscillations. For gel (12), chemical and mechanical oscillations were also visible and the ∆ϕ between them was about 0.1; the chemical oscillations occurred first, followed by the mechanical oscillations. The ∆ϕ (with the standard deviation, STD) and the values (∆L,%)(with the standard deviation, STD) of the amplitude of the chemomechanical oscillations for gels (8), (10), and (12) are listed in Table 1.

In other words, the polymer matrix of gel (12) reacted faster to the changes in the oxidation state of the catalysts than the matrix of gel (10), which indicated a different structure of the obtained gels.

**Figure 5:**
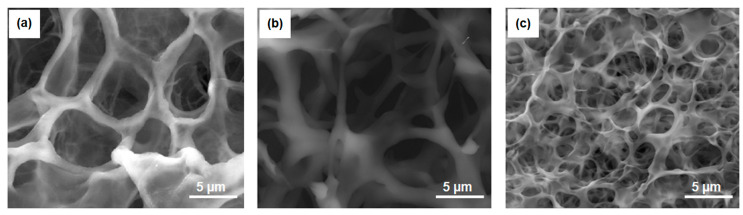
SEM images of polymer matrices of gels (8) (**a**), (10) (**b**), and (12) (**c**).

To study the influence of the structure of the gels on their chemomechanical properties, gels (8), (10), and (12) were analyzed via scanning electron microscopy (SEM) (Materials and Methods, Section 4.9). The structural differences between gels (8) and (10), containing a single catalyst [(3) or (6)], and gel (12), incorporating both catalysts [(3) and (6)], were distinctly observable in Figure 5. All the gels exhibited a disordered foam-like morphology. However, gels (8) and (10) presented a well-developed, large-scale mesh structure (Figure 5a,b). In contrast, gel (12) demonstrated a finer-scale polymer matrix (Figure 5c). The primary distinction between gels (8), (10), and gel (12) lay in the mesh size, which directly affected the packing density of the gels’ elastic components. To characterize the obtained gels, respectively, to the porosity of their structure, the approach of mathematical segmentation was applied to the wide-filed SEM images (Materials and Methods, Section 4.10).

**Figure 6:**
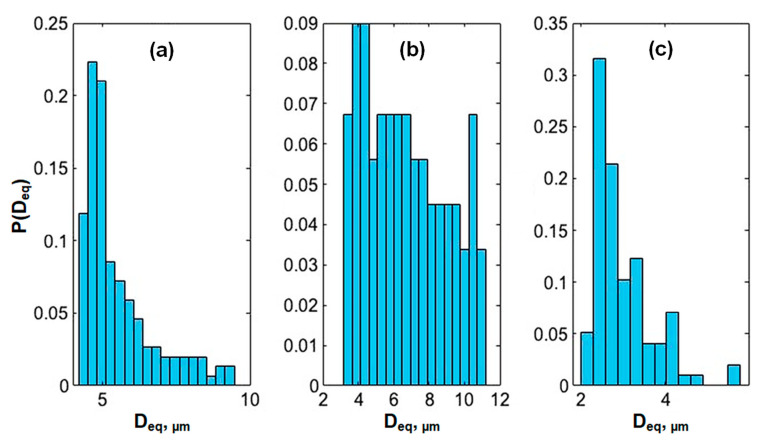
Histograms of the hole effective diameter distributions for gels 8 (**a**), 10 (**b**), and 12 (**c**). P(*D_eq_*) is the proportion of holes with an effective diameter *D_eq_*.

The resulting histograms of the hole size probability for gels (8), (10), and (12) are shown in Figure 6. The mean values E(*D_eq_*) with the standard deviations STD(*D_eq_*) are listed in Table 1.

As can be seen, the average hole effective diameters for gels (8) and (10) were 5.5 and 6.7 μm, respectively, while for gel (12) it was almost two times smaller (2.9 μm).

We also took measurements of the elastic properties of gels (8), (10), and (12) (a compressive load test, details see in Materials and Methods, Section 4.9). Respectively, the elastic compressive modulus (the Young modulus) was determined as E=σ/ϵ . The mean values (E) with the standard deviations (STDs) were listed in Table 1. As can be seen, the gel with a higher elastic modulus exhibited a larger amplitude of chemomechanical oscillations, and their correlation in a rough approximation could be expressed by the equation ∆L = 2.42E + 1.96.

It is well-established [23] that to achieve large mechanical oscillations relative to gel size, a self-oscillating gel with a rapid response to changes in the charge density of the metal catalyst in the Belousov–Zhabotinsky (BZ) reaction is required. We hypothesized that the oxidation [Ru(L_3_)^2+^→Ru(L_3_)^3+^] of the complexes had a more hydrating effect in gel (12) than in gels (8) and (10), since gel (12) had a 3–4 times more developed internal surface, the change in charge of which led to the faster absorption of liquid than in the case of gels (8) and (10). This led to an increase in the amplitude of the mechanical oscillations (11–16%) beyond the predicted value (8%), which was calculated by simply summing the concentrations of catalysts (3) and (6). Gels (8) and (10), with similar structures and comparable catalyst content, exhibited similar amplitudes of chemomechanical oscillations. In contrast, gel (12), with its distinct structure and greater sensitivity to changes in catalyst charge, combined with its higher catalyst content, resulted in a significant increase in the amplitude of the mechanical oscillations.

## 3. Conclusions

We synthesized the novel iron and ruthenium complexes of N4,N4′-bis[(3-(2-methylprop-2-enamido)propyl][2,2′-bipyridine]-4,4′-dicarboxamide, which served as catalysts for the oscillating Belousov–Zhabotinsky (BZ) reaction. Using these new catalysts as crosslinkers, we developed crosslinked poly(N-isopropylacrylamide) gels that exhibited self-oscillating chemical and mechanical behavior in the presence of the BZ reaction. Notably, we demonstrated for the first time that poly(N-isopropylacrylamide) gels crosslinked with iron complexes can exhibit chemical oscillations. The gels crosslinked with ruthenium complexes displayed swelling upon catalyst oxidation and shrinkage upon reduction, resulting in periodic changes of approximately 3.5% in linear size. We compared the self-oscillatory behavior and chemomechanical properties of the poly(N-isopropylacrylamide) gels crosslinked with iron and ruthenium complexes to those containing these complexes as pendant catalysts. Our findings revealed that gels with iron complexes, regardless of their role as pendant or crosslinking catalysts, showed different periodic changes in linear size (2% for pendant and 0% for crosslinking) at the same catalyst content. In contrast, the gels incorporating ruthenium complexes as either pendant or crosslinking catalysts exhibited similar periodic changes in linear size (4.5% and 3.5%, respectively) under identical catalyst loadings. For the first time, we have developed poly(N-isopropylacrylamide) self-oscillating gels that incorporate BZ catalysts as both crosslinkers and pendant fragments within the same gel matrix. The gels with iron catalysts demonstrated self-oscillating behavior without accompanying chemomechanical oscillations, whereas those with ruthenium catalysts exhibited high-amplitude autonomous chemomechanical oscillations, with linear size changes reaching up to 16%.

Our study revealed that the incorporation of both pendant and crosslinking ruthenium catalysts within the poly(N-isopropylacrylamide) gel resulted in a greater increase in the amplitude of the mechanical oscillations (11–16%) compared to the simple additive contributions of each catalyst (4.5% and 3.5%, respectively). Comparative studies of the microstructures and elastic properties of the synthesized gels demonstrated that the presence of both crosslinking and pendant catalysts in the same gel led to significant differences in the microstructure and elastic modulus when compared to the gels that were solely crosslinked or contained only pendant catalysts. The innovative aspect of this approach lay in the synergistic effect produced by combining different types of catalysts within a single gel matrix, which could result in substantial alterations to the gel’s structure and, consequently, a marked enhancement in the amplitude of the mechanical oscillations. This proposed methodology presents new avenues for the tailored modification of self-oscillating gel characteristics.

We believe that our approach opens up further prospects for the development of self-oscillating gels that exhibit significant mechanical oscillations and is suitable for the creation of chemomechanical devices, including self-actuating gel pumps and hydrogel-based motors.

## 4. Materials and Methods

### 4.1. Materials

For the synthesis, the following chemicals (all analytical grade, purchased from Aldrich, St. Louis, MO, USA) were used without further purification: ethyl acetate, acetone, methylene chloride, chloroform, benzene, methanol, ethanol, 2,2′-bipyridine-4,4′-dicarboxylic acid, N-(3-aminopropyl)methacrylamide hydrochloride, thionyl chloride, 2,2′-bipyridine, 1,10-phenanthroline, triethylamine, acryloyl chloride, 1,10-phenanthroline-5-amine, bis(2,2′-bipyridine)dichlororuthenium(II), iron(II) sulfate, dimethyl sulfoxide (DMSO), N-isopropylacrylamide (IPA), 2-acrylamido-2-methylpropanesulfonic acid (AMPS), N,N′-methylenebisacrylamide (MBA), ammonium persulfate (APS), tetramethylethylenediamine (TMEDA), malonic acid, sodium bromate, acetic acid, and distilled water. The chromatographic separation and purification of the compounds were performed using silica gel 60 (Aldrich) and Sephadex LH-20 (GE HealthCare Technologies, Inc., Chicago, IL, USA) as the sorbents.

The proton nuclear magnetic resonance (^1^H NMR) spectra of the synthesized compounds were recorded using a Bruker Avance III 400 MHz spectrometer (Bruker Corporation, Billerica, MA, USA) at 23 °C in Fourier transform mode. Chemical shifts (δ) were reported in parts per million (ppm) relative to the tetramethylsilane (TMS). The following abbreviations were used to describe the ^1^H NMR spectra: δ = chemical shift (ppm); J = spin–spin coupling constant (Hz); s = singlet; d = doublet; t = triplet; q = quartet; quint = quintet; m = multiplet; and dd = double doublet. The solvents (CD_3_)_2_SO, D_2_O, and CD_3_Cl were used for the ^1^H NMR studies. Elemental analyses were carried out using standard microanalysis techniques.

### 4.2. Synthesis of N4,N4′-bis[(3-(2-methylprop-2-enamido)propyl][2,2′-bipyridine]-4,4′-dicarboxamide (1)

The scheme of the synthesis of 1 was shown in Figure 1a. 2 2′-bipyridine-4 4′-dicarboxylic acid (2.5 mmol, 610.5 mg) was heated at 70 °C while stirring with thionyl chloride (137.7 mmol, 10 mL) for 2 days. The thionyl chloride was evaporated and chloroform (40 mL), N-(3-aminopropyl)methacrylamide hydrochloride (7.5 mmol, 1340 mg), and triethylamine (12.5 mmol, 1.738 mL) were added to the residue and left to stir at 25 °C for 2 days. Then, the mixture was evaporated, water (100 mL) and 28% ammonium solution (140 μL) were added, and the mixture was centrifuged. The sediment was washed with 100 mL of water and 10 mL of acetone. The substance was obtained in a 16% yield (191.4 mg = 0.4 mmol) as a white powder. The ^1^H NMR spectra in CD_3_Cl were as follows: δ 8.93 (t, J = 4.4 Hz, 2H, NH), 8.85 (d, J = 4.8 Hz, 2H, bpy-H), 8.77 (s, 2H, bpy-H), 7.93 (t, J = 5.2 Hz, 2H, NH), 7.83 (d, J = 4 Hz, 2H, bpy-H), 5.64 (s, 2H, C=CH_2_), 5.30 (s, 2H, C=CH_2_), 3.30–3.20 (m, 4H, CH_2_), 3.70 (q, J = 6.4 Hz, 4H, CH_2_), 1.84 (s, 6H, CH_3_), and 1.72 (quint, J = 6.8 Hz, 4H, CH_2_). The elemental analysis data for C_26_H_32_N_6_O_4_ × H_2_O (%) were as follows: calculated C 61.16, H 6.71, N 16.46, and O 15.67; found C 60.93, H 6.53, and N 15.42.

### 4.3. Synthesis of (N4,N4′-bis[(3-(2-methylprop-2-enamido)propyl][2,2′-bipyridine]-4,4′-dicarboxamide)bis(2,2′-bipyridine)iron(II) Sulfate (2)

The scheme of synthesis of (2) is shown in Figure 1a. To synthesize (2), 2,2′-bipyridine (31.2 mg = 0.2 mmol) and (1), (49.3 mg = 0.1 mmol) were dissolved in 5 mL of methanol and the resulting mixture was stirred with 100 µL of 1 M solution of iron(II) sulfate (15.2 mg = 0.1 mmol). The obtained mixture was stirred within 1 h at 25 °C and evaporated under the reduced pressure. The red residue was dissolved in 2 mL of H_2_O; unreacted compounds were extracted 2 times with 2 mL of benzene, and the complex was purified via column chromatography (Sephadex LH-20, H_2_O). The water solution was evaporated under reduced pressure to give (2) (76.6 mg = 0.08 mmol) as a red powder with the 80% yield. The ^1^H NMR spectra in D_2_O were as follows: δ 8.82–8.78 (m, 2H, bpy-H), 8.46–8.41 (m, 4H, bpy-H), 8.02–7.94 (m, 6H, bpy-H), 7.58–7.52 (m, 4H, bpy-H), 7.38–7.32 (m, 4H, bpy-H), 7.29–7.21 (m, 4H, bpy-H), 5.50 (s, 2H, C=CH_2_), 5.20 (s, 2H, C=CH_2_), 3.38 (t, J = 6.4 Hz, 4H, CH_2_), 3.25 (t, J = 6.0 Hz, 4H, CH_2_), 1.79–1.76 (m, 4H, CH_2_), and 1.71 (s, 6H, CH_3_). The elemental analysis data for C_46_H_48_N_10_O_8_SFe × 6H_2_O (%) were as follows: calculated C 51.88, H 5.68, and N 13.15; found C 51.87, H 5.26, and N 13.08. The maxima λ_max_ (nm) of the UV-visible spectra in water (and the corresponding molar extinction coefficients in units M^−1^⋅cm^−1^) were as follows: 201 (59,820), 247 (30,982), 298 (49,606), 350 (6814), and 523 (9276).

### 4.4. Synthesis of (N4,N4′-bis[(3-(2-methylprop-2-enamido)propyl][2,2′-bipyridine]-4,4′-dicarboxamide)bis(2,2′-bipyridine)ruthenium(II) Chloride (3)

To synthesize (3), (Figure 1a), bis(2,2′-bipyridine)dichlororuthenium(II) (48.4 mg, 0.1 mmol) and (1) (49.3 mg, 0.1 mmol) were mixed in 2 mL of ethanol. The mixture was intensively shaken for 11 h at 70 °C, cooled to room temperature, and filtered. The filtrate was evaporated under the reduced pressure. The orange-red residue was dissolved in 5 mL of H_2_O and the unreacted compounds were extracted 2 times with 5 mL of chloroform. The water solution was evaporated under reduced pressure to give (3) (72.3 mg, 0.074 mmol) as an orange-red powder in 74% yield. The ^1^H NMR spectra in D_2_O were as follows: δ 8.76 (s, 2H, bpy-H), 8.42 (d, J = 8.0 Hz, 4H, bpy-H), 7.94 (t, J = 8.0 Hz, 4H, bpy-H), 7.88 (d, J = 6.0 Hz, 2H, bpy-H), 7.67 (d, J = 6.0 Hz, 2H, bpy-H), 7.63 (d, J = 5.2 Hz, 2H, bpy-H), 7.53 (dd, J = 6.0 Hz, J = 1.2 Hz, 2H, bpy-H), 7.26 (t, J = 6.8 Hz, 4H, bpy-H), 5.49 (s, 2H, C=CH_2_), 5.19 (s, 2H, C=CH_2_), 3.37 (t, J = 6.4 Hz, 4H, CH_2_), 3.24 (t, J = 6.4 Hz, 4H, CH_2_), 1.77 (quint, J = 6.8 Hz, 4H, CH_2_), and 1.70 (s, 6H, CH_3_). The elemental analysis data for C_46_H_48_Cl_2_N_10_O_4_Ru × 6H_2_O (%) were as follows: calculated C 50.92, H 5.57, and N 12.91; found C 50.89, H 4.56, and N 12.94. The maxima λ_max_ (nm) of the UV-visible spectra in water (and the corresponding molar extinction coefficients in units M^−1^⋅cm^−1^) were as follows: 195 (67,100), 245 (33,937), 286 (56,070), 361 (9310), 477 (11,965).

### 4.5. Synthesis of 5-Acrylamide-1,10-phenanthroline (4)

The ligand (4) was synthesized as described in the previous work [9]. Tetramethylethylenediamine (29.9 μL, 23.2 mg = 0.200 mmol) and 1,10-phenanthroline-5-amine (58.6 mg = 0.300 mmol) were mixed in 9 mL of dry tetrahydrofuran. The obtained suspension was stirred within 1 h at 10 °C and a solution of acryloyl chloride (28.5 μL, 31.9 mg = 0.352 mmol) in 1 mL of tetrahydrofuran was added. The mixture obtained was stirred for 20 h at 10 °C. Then, tetrahydrofuran was evaporated under reduced pressure and (4) was isolated from the residue via column chromatography (silica gel, ethanol–methylene chloride 1:7). The yield was about 49% (37.0 mg = 0.148 mmol). The ^1^H NMR spectra in (CD_3_)_2_SO were as follows: δ 10.33 (s, 1H, NH), 9.13 (d, J = 2.8 Hz, 1H, phen-H), 9.03 (d, J = 4 Hz, 1H, phen-H), 8.60 (dd, J = 8 Hz, J = 0,8 Hz, 1H, phen-H), 8.45 (dd, J = 8 Hz, J =1,2 Hz, 1H, phen-H), 8.29 (s, 1H, phen-H), 7.87–7.78 (m, 1H, phen-H), 7.77–7.68 (m, 1H, phen-H), 6.79–6.64 (m, 1H, CH=CH_2_), 6.35 (d, J = 16.8 Hz, 1H, CH=CH_2_), and 5.86 (d, J = 10.4 Hz, 1H, CH=CH_2_). The elemental analysis data for C_15_H_11_N_3_O (%) were as follows: calculated C 72.28, H 4.45, and N 16.86; found C 72.31, H 4.50, and N 16.83.

### 4.6. Synthesis of (5-Acrylamido-1,10-phenanthroline)bis(1,10-phenanthrolin)iron(II) Sulfate (5)

The scheme of the synthesis of (5) is shown in Figure 1b. To synthesize (5), (4) (24.9 mg = 0.1 mmol) and 1,10-phenantroline (36.4 mg = 0.2 mmol) were mixed in 5 mL of methanol and the resulting solution was stirred with 100 µL of 1 M solution of iron(II) sulfate (15.2 mg = 0.1 mmol). The mixture was stirred within 1 h at 25 °C and evaporated under reduced pressure. The obtained residue was dissolved in 2 mL of H_2_O and the unreacted compounds were extracted 2 times with 2 mL of benzene. The water solution was evaporated under reduced pressure to give (5) (61 mg = 0.8 mmol) as a red powder with an 80% yield. The ^1^H NMR spectra in D_2_O were as follows: δ 8.55–8.45 (m, 6H, phen-H), 8.20–8.10 (m, 5H, phen-H), 7.65–7.60 (m, 6H, phen-H), 7.50–7.45 (m, 6H phen-H), 6.56 (t, 1H, J = 8.14 Hz, CH=CH_2_), 6.37 (d, J = 8.14 Hz, 1H, CH=CH_2_), and 6.93 (d, J = 8.15 Hz, 1H, CH=CH_2_). The elemental analysis data for C_39_H_27_N_7_O_5_SFe × 3H_2_O (%) were as follows: calculated C 57.43, H 4.08, and N 12.02; found C 57.35, H 4.28, and N 11.88. The maxima λ_max_ (nm) of the UV-visible spectra in water (and the corresponding molar extinction coefficients in units M^−1^⋅cm^−1^) were as follows: 228 (44,000), 254 (37,040), and 511 (11,092).

### 4.7. Synthesis of Bis(2,2′-bipyridine)(5-acrylamido-1,10-phenanthroline)ruthenium(II) Chloride (6)

To synthesize (6) (Figure 1b), 5-acrylamido-1,10-phenanthroline (123.6 mg, 0.496 mmol) and bis(2,2′-bipyridine)dichlororuthenium(II) (200.0 mg, 0.413 mmol) were mixed in 10 mL of ethanol. The mixture was stirred for 11 h at 70 °C. Then, the reaction mixture was cooled to room temperature and filtered from bis(2,2′-bipyridine)dichlororuthenium(II); the filtrate was evaporated under reduced pressure. The orange-red residue was dissolved in 5 mL of H_2_O and unreacted 5-acrylamido-1,10-phenanthroline was extracted 2 times with 10 mL of ethyl acetate and 2 times with 10 mL of methylene chloride. The water solution was evaporated under reduced pressure to give (6) (206.0 mg, 0.281 mmol) as an orange-red powder in 68% yield. The ^1^H NMR spectra in D_2_O were as follows: δ 10.86 (s, 1H, NH), 9.03 (d, J = 8.4 Hz, 1H, phen-H), 8.9–8.79 (m, 4H, phen-H), 8.77 (s, 1H, phen-H), 8.74 (d, J =8.0 Hz, 1H, phen-H), 8.20 (t, J = 8.0 Hz, 2H, bpy-H), 8.15 (d, J = 7.2 Hz, 1H, bpy-H), 8.09 (td, J = 7.7 Hz, J = 2.8 Hz, 2H, bpy-H), 8.02 (dd, 1H, J = 5.2 Hz, bpy-H), 7.90 (m, 1H, bpy-H), 7.81 (t, J = 6.6 Hz, 3H, bpy-H), 7.56 (d, J = 5.2 Hz, 4H, bpy-H), 7.35 (t, J = 6.6 Hz, 2H, bpy-H), 6.89 (dd, J = 16.5 Hz, J = 10.2 Hz,1H, CH=CH_2_), 6.40 (d, J = 16.8 Hz, 1H, CH=CH_2_), and 5.92 (d, J = 11.6 Hz, 1H, CH=CH_2_). The elemental analysis data for C_35_H_27_N_7_Cl_2_ORu × H_2_O (%) were as follows: calculated C 55.93, H 3.89, N 13.04; found C 55.90, H 3.91, and N 12.97. The maxima λ_max_ (nm) of the UV-visible spectra in water (and the corresponding molar extinction coefficients in units M^−1^⋅cm^−1^) were as follows: 192 (67,192), 244 (36,898), 285 (63,267), and 451 (13,915).

### 4.8. Synthesis of Gels (7)–(12)

To synthesize gels (7)–(12), we prepared pre-gels (472 μL), which were solutions of IPA, AMPS, and MBA, and catalysts in 53% aqueous DMSO in the concentrations indicated in Table 2. The resulting pre-gels were degassed under a vacuum and heated to 70 °C. To start polymerization, 14 μL of 1M TMEDA (1.6 mg = 0.014 mmol) and 14 μL of 1M ammonium persulfate (APS) (3.2 mg = 0.014 mmol) in water were added to each pre-gel. The mixtures obtained were heated at 70 °C for 1 h. The resulting gels (7)–(12) were incubated 3 times for 1 day in 10 mL of distilled water to remove the unreacted low-molecular compounds.

The amount (N, mmol) of catalysts in the resulting gels (see in Section 2.1, Section 2.2 and Section 2.3) was determined as the difference between the amount of catalysts (N_loaded_) introduced during synthesis and the amount of catalysts (N_washed out_) washed out by rinsing the finished gel with water (N_inside_ = N_loaded_ **−** N_washed out_). The value N_washed out_ was estimated spectrophotometrically (N_washed out_ = C_washed out_ × V_washed out_; C_washed out_ = A_catalyst_/ε_catalyst_). Since the gel periodically changed volume, the amount of the catalyst inside the gel was referred to the amount of the main monomer IPA (mol%_catalyst_ = N_catalyst_/IPA) and not to the volume of the gel.

### 4.9. Experimental Setup

Oscillations of the pieces of gels (7), (9), (11), immersed in the CFBZ solution (=catalyst free BZ solution) were observed using a microscope (Zeiss Stemi-2000, Carl Zeiss Microscopy GmbH, Oberkochen, Germany) equipped with a color camera (AxioCam ERc5s, Carl Zeiss Microscopy GmbH, Oberkochen, Germany) connected to a personal computer; oscillations of the pieces of gels (8), (10), (12) were observed using a microscope (Zeiss Stemi-2000) equipped with a black-white CCD camera (QImaging Retiga 2000R, QImaging, Surrey, BC, Canada) connected to a personal computer. A Petri dish with pieces of gels (7), (9), (11) was illuminated from below with a LED light source. A Petri dish with pieces of gels (8), (10), (12) was illuminated from below with a LED light source through an interference filter with the wavelength of the maximum transmission at λ = 450 nm. The recording of light transmission through the pieces of the gels and the geometrical measurements of the pieces of the gels have been performed using software (QCapture ProS2, QImaging, Surrey, BC, Canada). For each piece of gel, the space–time plot was constructed for cross-sections indicated by arrows (Figure 3). Cross-sections through the stack of the pictures were made to reduce the dimensionality into one spatial dimension and time. The sequence of the cross-sections (recorded every second) in time was combined together to create a space–time plot for the analysis of the linear dimensions and the periods of the oscillations. SEM images of the gels were obtained using the scanning electron microscope Quanta FEG 650 (Thermo Fisher Scientific, Waltham, MA, USA), applying a method of cryofixation and drying it in a vacuum in the setup’s chamber. Samples of the gels were prepared as follows: small pieces of the gels, about 1 mm, were placed in liquid nitrogen for 1 min until completely frozen, then put in the microscope’s chamber with a low vacuum for drying.

Measurements of elastic properties of gels (a compressive load test) were carried out using the experimental setup, which combined the electromagnetic force balancing system Vibra XFR-205DRE (Shinko Denshi Co., Ltd.; Shimotsuma-city, Japan) and the precision length gauge IZV-2 (LOMO; Moscow, Russia).

### 4.10. Data Processing

To improve visualization, the mean pixel intensity within the gel region was plotted, particularly when color changes were subtle. A Python-based algorithm, utilizing core functions from the ‘OpenCV’ library, was developed to convert the video footage into corresponding intensity graphs. The code was executed in JupyterLab (open-source software, v4.2.5), allowing for the interactive analysis of the data. The algorithm operated in two key phases for each frame of the video. First, computer vision techniques were applied to distinguish the gel from the background. Segmentation was performed in the HSV color space by setting specific thresholds for the hue and saturation channels, based on their respective color histograms. In the second phase, the average brightness of the segmented region was calculated using the value (V) channel, which represented pixel intensity.

To characterize the porosity of the gels, the approach of mathematical segmentation was applied to wide-filed SEM images using the ’MorphoLibJ’ plugin [24] for ImageJ bundled with Zulu OpenJDK 13.0.6 (Fiji) software. At the first step, following the standard workflow of mathematical morphology and segmentation [25], Gaussian filtering aimed at eliminating the ridges of low intensity visible through the surface holes, was carried out. It was followed by threshold-based binarization with subsequent smoothing to obtain an image depicting a well-bounded hole structure, which allowed for sable gradient edge detection. The correspondence between the revealed structure of the hole distributions and the porosity seen in the raw image was controlled by the applying superposed semi-opaque overlays using the standard tools of ImageJ (Fiji).

For such preprocessed images, the scaled area and circularity of the detected features corresponding to the holes were determined. Circularity was defined in a standard way as *C* = *πS/L*^2^, where *S* and *L* were the spot’s area and perimeter, and was used to exclude objects corresponding to polymer filaments misinterpreted as hole-like structures; the objects with circularities falling into the lowest 5% quantile of the circularity distribution as well as those which had *C* > 1 were eliminated from further consideration. To characterize the typical sizes of hundreds of the rest ones, we determined the standard quantitative measure used for this goal [26]; the equivalent diameter was defined as D _eq_ $=\sqrt{S/\pi }$ , i.e., as the diameter of the disk having the same area. The resulting histograms of the hole size probability are shown in Figure 6. The mean values E (D_eq_) with the standard deviations, STDs (D_eq_), are listed in Table 1.

## Figures and Tables

**Table 1:** Characteristics of gels (8), (10), and (12).

Gel	8	10	12
∆ϕ	-	0.15	0.1
STD	-	0.01	0.01
∆L, %	3.5	4.5	11(16)
STD, %	0.4	0.5	1
E(*D_eq_*), µm	5.5	6.7	2.9
STD(*D_eq_*), µm	1.2	2.3	0.71
E, kPa	0.43	1.35	3.67
STD, kPa	0.15	0.32	0.59

**Table 2:** Composition of pre-gels for the synthesis of gels (7)–(12). Component concentrations are given in millimoles per liter (mM).

Gel	IPA	AMPS	MBA	2	3	5	6
7	1380	28	0	20	0	0	0
8	1380	28	0	0	20	0	0
9	1380	28	14	0	0	20	0
10	1380	28	14	0	0	0	20
11	1380	28	0	20	0	20	0
12	1380	28	0	0	20	0	20

## Data Availability

The raw/processed data required to reproduce these findings cannot be shared at this time as the data also form part of an ongoing study.

## Supplementary Materials

The following supporting information can be downloaded at https://www.mdpi.com/article/10.3390/gels10110727/s1, Movie S1, “Piece of gel 12 (sped up 64 times)”, and Movie S2, “Peristaltic motion of gel 12 (sped up 64 times)”.
