# Correction: Mallphanov et al. Novel Approach to Increasing the Amplitude of the Mechanical Oscillations of Self-Oscillating Gels: Introduction of Catalysts Both as Pendant Groups and as Crosslinkers. *Gels* 2024, *10*, 727

**DOI:** 10.3390/gels10120830

**Published:** 2024-12-16

**Authors:** Ilya L. Mallphanov, Michail Y. Eroshik, Dmitry A. Safonov, Alexander V. Sychev, Vyacheslav E. Bulakov, Anastasia I. Lavrova

**Affiliations:** 1 Center for Nonlinear Chemistry, Immanuel Kant Baltic Federal University, 14 A. Nevskogo Street, Kaliningrad 236016, Russia; misha.eroshik@yandex.ru (M.Y.E.); dasafonoff@gmail.com (D.A.S.); aurebours@googlemail.com (A.I.L.); 2 Research Center for Condensed Matter Physics, Kursk State University, 33 Radishcheva Street, Kursk 305000, Russia; sychev1113@gmail.com; 3 Department of General and Bioorganic Chemistry, Pavlov First Saint-Petersburg State Medical University, 6-8 L’va Tolstogo Street, Saint-Petersburg 197022, Russia; bulakov_v@mail.ru; 4 Saint-Petersburg State Research Institute of Phthisiopulmonology, 2-4 Ligovsky Avenue, Saint-Petersburg 191036, Russia

## Errors in Figure 1

In the original publication [1], there were mistakes in Figure 1 as published. In Figure 1 there were errors in the reaction conditions (partially incorrect captions near the arrows). The corrected Figure 1 appears below. Also, due to the changes in Figure 1, we ask that the caption to it be changed from Figure 1. (**a**) Synthesis of ligand (1) and catalysts (2) and (3). (**b**) Synthesis of ligand (4) and catalysts (5) and (6). TEA—triethylamine, Ru(bpy)_2_Cl_2_—bis(2,2′-bipyridine)ruthenium(II) chloride, EtOH—ethanol. to Figure 1. (**a**) Synthesis of ligand (**1**) and catalysts (**2**) and (**3**). (**b**) Synthesis of ligand (**4**) and catalysts (**5**) and (**6**). TEA—triethylamine, TMEDA—tetramethylethylenediamine, Ru(bpy)_2_Cl_2_—bis(2,2′-bipyridine)ruthenium(II) chloride, THF—tetrahydrofuran, MeOH—methanol, EtOH—ethanol.

The authors state that the scientific conclusions are unaffected. This correction was approved by the Academic Editor. The original publication has also been updated.

**Figure 1:**
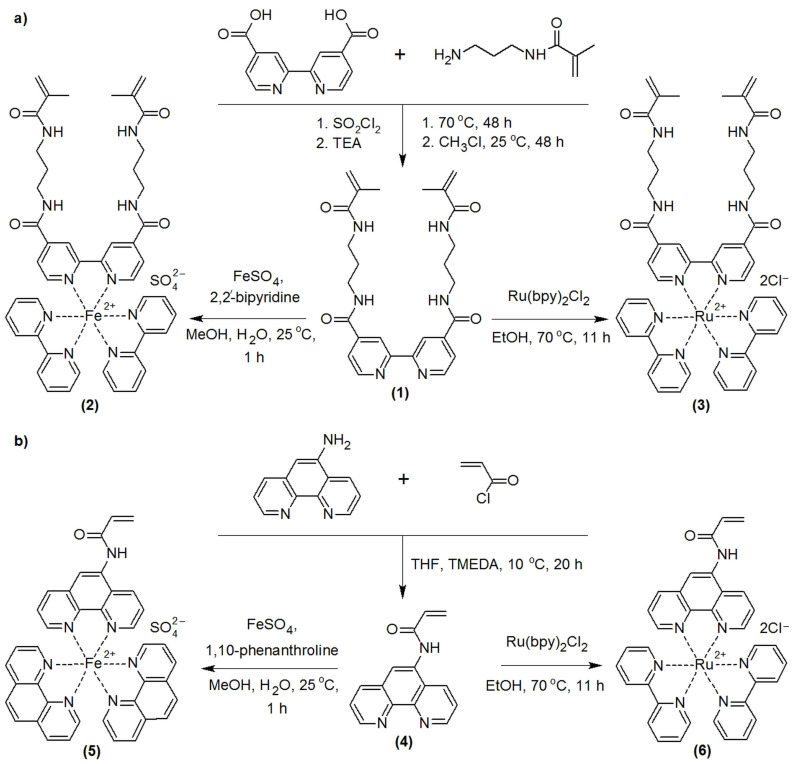
(**a**) Synthesis of ligand (**1**) and catalysts (**2**) and (**3**). (**b**) Synthesis of ligand (**4**) and catalysts (**5**) and (**6**). TEA—triethylamine, TMEDA—tetramethylethylenediamine, Ru(bpy)_2_Cl_2_—bis(2,2′-bipyridine)ruthenium(II) chloride, THF—tetrahydrofuran, MeOH—methanol, EtOH—ethanol.
